# Behaviors of *Microcystis aeruginosa* cells during floc storage in drinking water treatment process

**DOI:** 10.1038/srep34943

**Published:** 2016-10-07

**Authors:** Hangzhou Xu, Haiyan Pei, Hongdi Xiao, Yan Jin, Xiuqing Li, Wenrong Hu, Chunxia Ma, Jiongming Sun, Hongmin Li

**Affiliations:** 1 School of Environmental Science and Engineering, Shandong University, Jinan, 250100, China; 2 Shandong Provincial Engineering Center on Environmental Science and Technology, Jinan, 250061, China; 3 School of Physics, Shandong University, 250100, China

## Abstract

This is the first study to systematically investigate the different behaviors of *Microcystis aeruginosa* in the sludges formed by AlCl_3_, FeCl_3_, and polymeric aluminium ferric chloride (PAFC) coagulants during storage. Results show that the viability of *Microcystis aeruginosa* in PAFC sludge was stronger than that of cells in either AlCl_3_ or FeCl_3_ sludge after the same storage time, while the cells’ viability in the latter two systems stayed at almost the same level. In AlCl_3_ and FeCl_3_ sludges high concentrations of Al and Fe were toxic to *Microcystis aeruginosa*, whereas in PAFC sludge low levels of Al showed little toxic effect on *Microcystis aeruginosa* growth and moderate amounts of Fe were beneficial to growth. The lysis of *Microcystis aeruginosa* in AlCl_3_ sludge was more serious than that in PAFC sludge, for the same storage time. Although the cell viability in FeCl_3_ sludge was low (similar to AlCl_3_ sludge), the *Microcystis aeruginosa* cells remained basically intact after 10 d storage (similar to PAFC sludge). The maintenance of cellular integrity in FeCl_3_ sludge might be due to the large floc size and high density, which had a protective effect for *Microcystis aeruginosa*.

The presence of toxic cyanobacteria in natural waters poses a threat to animal and human health because they can produce many types of toxic compounds^1^,^2^, which can cause serious and even fatal human liver, digestive, neurological, and skin diseases^2^,^3^. Besides this, the water quality can also be greatly diminished, for example, the aesthetics of drinking water can be compromised by taste and odor compounds produced by cyanobacteria such as geosmin and 2-methylisoborneol. Recently, due to nutrient over-enrichment of surface waters by urban, agricultural, and industrial development, cyanobacterial blooms have quickly become a global epidemic, including Lake Victoria in Africa, Lake Winnipeg in Canada, Lake Taihu in China, and the Baltic Sea in Europe^4^,^5^,^6^,^7^. Although most of the cyanotoxins are present within cells^8^,^9^ - with the exception of *Cylindrospermopsis sp.*, which can have a large amount of the toxin extracellularly^10^,^11^ - the intracellular toxins can be released into surrounding waters under certain external stresses responsible for cell lysis. The release of intracellular toxins poses a more serious hazard to water safety because of their more difficult removal compared to the whole cells^12^,^13^. Therefore, it has become very important to find an effective method of removing cyanobacteria without cell damage.

Coagulation, the key step in conventional drinking water treatment for pollutant removal, has been verified as an effective approach to remove intracellular toxins with intact cells, without causing additional release of intracellular toxins^12^,^14^. The successful removal of intact algal cells can avoid the release of intracellular material, especially the toxins, into the supernatant, decreasing the burden on subsequent processes and decreasing production of toxic disinfection byproducts^15^,^16^. After coagulation/flocculation and sedimentation processes, the intact cyanobacteria cells are transferred into a solid phase, namely the drinking water sludge.

Large amounts of drinking water sludge will be produced during the production of drinking water, equivalent to 4–7% of the total drinking water produced^17^,^18^. Furthermore, due to the global water scarcity in recent years, more and more drinking water sludge is dewatered, with the extracted water being recycled into the production stream. However, in the treatment of cyanobacteria-containing drinking water sludge, damage to cyanobacterial cells will result in the release of intracellular material, especially toxins, into the recycled water, thus increasing the burden of toxin removal^17^. Therefore, knowledge of cell viability, integrity and intracellular metabolite release during the sludge treatment process is required. How do cyanobacteria cells behave when transferred to the sludge? Up to now, little attention has been drawn to it.

Based on our previous study, cyanobacteria cells coagulated by the conventional coagulants (including AlCl_3_, FeCl_3_, and PAFC) lysed on different days during floc storage^19^,^20^,^21^, which means that there may be different responses or behavior of cyanobacteria present in the various sludges. Hence, in this study we will systematically explore the trends of cell viability and integrity of cyanobacteria in the sludge, and then reveal the possible mechanisms of these processes.

## Results and Discussion

### Effects of coagulant species on *M. aeruginosa* cell integrity during sludge storage

**Figure 1:**
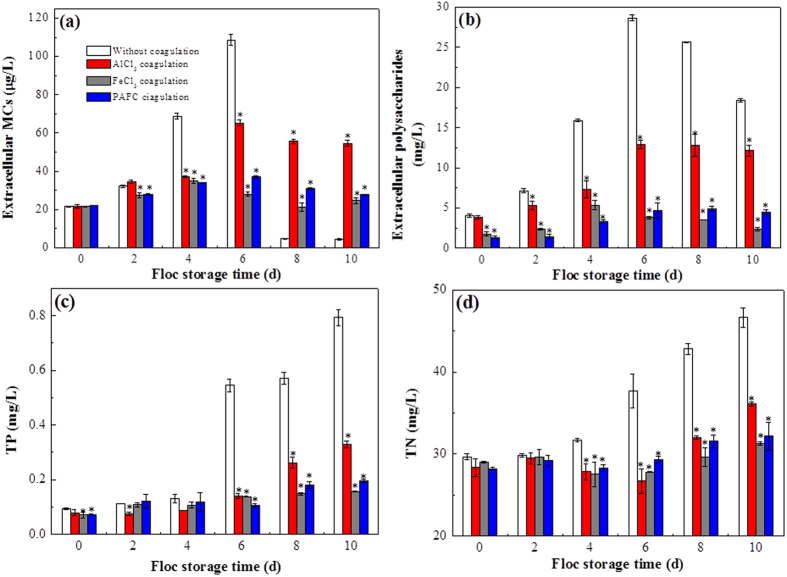
Extracellular concentrations of (**a**) MCs, (**b**) polysaccharide, (**c**) TP, and (**d**) TN after storage for 0, 2, 4, 6, 8, and 10 d. Data are shown as the mean ± SD (n = 3). Asterisks above the bars indicate significant differences with respect to the system without coagulation (*P* < 0.05).

To investigate the cell integrity of *M. aeruginosa* during the sludge storage process, we analyzed the changes of extracellular microcystins (MCs), polysaccharide, TP, and TN after storage for 0, 2, 4, 6, 8, and 10 d, and the results are given in Fig. 1(a–d). The extracellular MCs concentration in the four systems (without coagulation, AlCl_3_, FeCl_3_, and PAFC coagulation, respectively) initially increased and then declined with prolonged storage time, however, the variation of the extracellular MCs in the system without coagulation was more obvious than that in the systems with coagulation (Fig. 1(a)). The initial increase in the extracellular MCs may be due to the secretion of MCs by natural processes^22^, or the release of intracellular MCs from the broken *M. aeruginosa* cells. Furthermore, the extracellular MCs concentration reached 108.5 ± 2.9 μg/L (the maximum level) on the sixth day in the system without coagulation, which means that the lysis of *M. aeruginosa* cells might occur and most of the intracellular MCs were released out after 6 d storage. For the systems with coagulation, the extracellular MCs were lower than that in the system without coagulation (*P* < 0.05). The main reason may be that the algal cells can be coated with a layer of coagulant owing to the charge neutralization between the negatively charged cells and the positively charged coagulant, which may have inhibited the release of intracellular substances and prevented the lysis of algae cells to some extent^19^,^20^,^23^,^24^. Another potential reason may be that the extracellular polymeric substances produced by *M. aeruginosa* cells increased due to the exposure of cells to external stimulus, and thus the protective shield formed by extracellular polymeric substances was strengthened for cells responding to the external stress^20^. In addition, the extracellular MCs concentration of cyanobacteria-containing AlCl_3_ sludge increased gradually during days 4–6, then decreased slightly during days 8–10. The extracellular MCs concentrations of cyanobacteria-containing FeCl_3_ and PAFC sludges had no significant increase during sludge storage, indicating that the damage of the cells in AlCl_3_ sludge is higher than that in FeCl_3_ and PAFC sludge. Besides this, the extracellular MCs levels decreased from 108.5 ± 2.9 μg/L (the sixth day) to 4.7 ± 0.1 μg/L within 2 d in the system without coagulation, which might be due to the biodegradation by bacteria. Rapid degradation of MCs by coexisting bacteria of *M. aeruginosa*, such as *Bacillus cereus, Burkholderia* sp. and *Pseudomonas* sp., in natural lakes, reservoirs, and laboratory culture has been reported^25^,^26^,^27^. In addition, despite decline in the level of extracellular MCs for AlCl_3_, FeCl_3_, and PAFC sludges upon prolonged storage, the remaining MC concentrations were still 54.6 ± 1.5, 24.7 ± 1.5, and 27.8 ± 0.3 μg/L after 10 d storage. Even though extracellular MCs could be degraded by the aforementioned bacteria, the slightly damaged algal cells could release intracellular MCs outside gradually during the whole storage process. The combination of these two opposing effects may account for the observation that extracellular MCs remained at a relatively high level for up to 10 d of storage.

Figure 1(b) shows the levels of extracellular polysaccharide during the storage time. The extracellular polysaccharide concentration in the four systems also initially increased and then declined with increasing storage time, and the degree of change in the extracellular polysaccharide in the system without coagulation was higher than that in the systems with coagulation (*P* < 0.05), which is a similar trend to that seen with the extracellular MCs (Fig. 1(a)). As the storage time increased, the extracellular polysaccharide reached the maximum value of 28.7 ± 0.3 mg/L in the uncoagulated system on the sixth day, while the extracellular polysaccharide only reached 12.9 ± 0.5 mg/L at the same storage time in the cyanobacteria-containing AlCl_3_ sludge. Furthermore, there was no obvious increase of extracellular polysaccharide in the cyanobacteria-containing FeCl_3_ and PAFC sludge during the sludge storage period. These results were consistent with the results of extracellular MCs described above.

As storage time increased, the concentrations of TP in the four systems increased (see Fig. 1(c)). Moreover, the TP level in the system without coagulation rose significantly from 0.13 ± 0.01 mg/L on the fourth day to 0.55 ± 0.02 mg/L on the sixth day, and then increased to 0.79 ± 0.03 mg/L after 10 d storage. This result also illustrated that the damage of *M. aeruginosa* cells began to occur on the sixth day and the lysis of *M. aeruginosa* cells may be more serious with prolonged storage. Compared to the system without coagulation, the concentrations of TP were 0.33 ± 0.01, 0.17 ± 0.01, and 0.2 ± 0.01 mg/L in the cyanobacteria-containing AlCl_3_, FeCl_3_ and PAFC sludge after 10 d storage, respectively, which showed that (i) the flocs had the protection of *M. aeruginosa* cells, (ii) the lysis of algal cells in AlCl_3_ sludge was higher than the cells in FeCl_3_ and PAFC sludges, and (iii) there was basically no breakage of *M. aeruginosa* cells in FeCl_3_ and PAFC sludge. In addition, the variation of TN in Fig. 1(d) also showed a similar trend, and further verified the results above.

**Figure 2:**
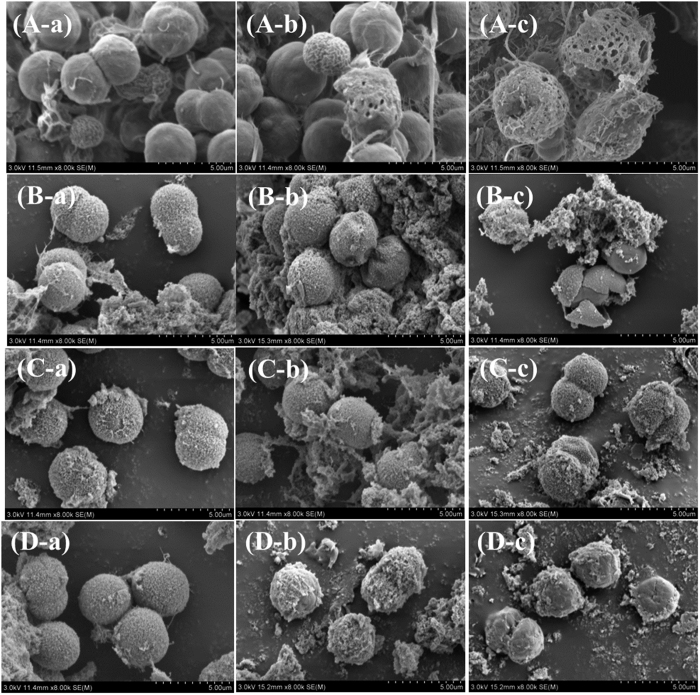
SEM micrographs of *M. aeruginosa* cells before and after coagulation. (**A**) Cells with no coagulant, (**B**) cells with AlCl_3_ coagulation, (**C**) cells with FeCl_3_ coagulation, (**D**) cells with PAFC coagulation. Flocs were stored for (a) 0 d, (b) 6 d, (c) 8 d.

As previously reported, SEM has been an intuitive and indispensable tool for the estimation of cell damage in recent years^28^. It is a visual method that images a sample producing signals that contain information about the sample’s surface topography, composition, and other properties^29^. Thus, to further verify the above inference, four systems were established to directly detect the *M. aeruginosa* cell changes in different conditions: (A) without coagulation, (B) AlCl_3_ coagulation, (C) FeCl_3_ coagulation, and (D) PAFC coagulation, and after the cells were stored for (a) 0 d, (b) 6 d, and (c) 8 d. The samples in these systems were taken for SEM analysis, as shown in Fig. 2. For the system without coagulation, the *M. aeruginosa* cells were intact and the surfaces were smooth with 0 d storage (see Fig. 2(A-a)), however, the *M. aeruginosa* cells began to lyse after 6 d (Fig. 2(A-b)) and were almost completely damaged after 8 d (Fig. 2(A-c)). Although the *M. aeruginosa* cells remained intact after 6 d storage following AlCl_3_ coagulation, some cells were no longer spherical and seemed to have collapsed (Fig. 2(B-b)), and some *M. aeruginosa* cells were damaged when stored for 8 d (Fig. 2(B-c)). In addition, although some collapse was found on the surface of the FeCl_3_-coagulated *M. aeruginosa* cells after 8 d storage (Fig. 2(C-c), the *M. aeruginosa* cells remained intact and no lysis occurred until 8 d in the systems with FeCl_3_ and PAFC coagulation (Fig. 2(C-c,D-c)). Therefore, the lysis of *M. aeruginosa* cells varied in the following sequence for a given storage time: *M. aeruginosa* without coagulation > AlCl_3_ sludge > FeCl_3_ sludge ≈ PAFC sludge, which was consistent with the variations of extracellular MCs, polysaccharide, TP, and TN described above.

### Effects of coagulant species on *M. aeruginosa* cell viability during sludge storage

**Figure 3:**
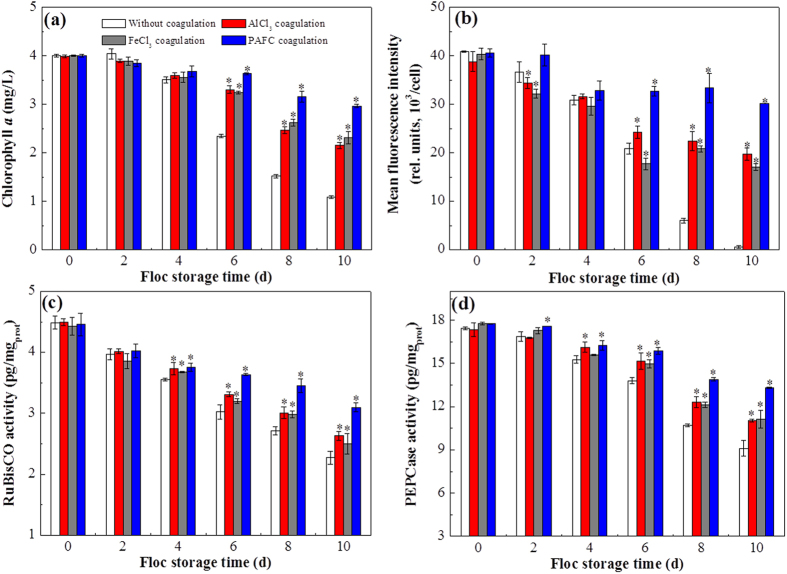
Chlorophyll *a* concentrations (**a**), mean chlorophyll *a* autofluorescence intensity (**b**), RuBisCO (**c**), and PEPCase activity (**d**) in the four systems at different floc storage times (0, 2, 4, 6, 8, and 10 d). Data are shown as the mean ± SD (n = 3). Asterisks above the bars indicate significant differences with respect to the system without coagulation (*P* < 0.05).

The cell viability of *M. aeruginosa* cells in sludge represents the risk of *M. aeruginosa* cells regrowth. Therefore, the effect of coagulant species on *M. aeruginosa* cell viability during sludge storage was investigated so as to inform the safe treatment of coagulation sludge in drinking water treatment plants. According to our previous study, there was a strong linear relationship between the chlorophyll *a* concentration and cell viability for *M. aeruginosa* cells^20^. Thus, chlorophyll *a* concentration served as an index of *M. aeruginosa* cell viability in our study. The changes of chlorophyll *a* in the systems without coagulation and with AlCl_3_, FeCl_3_ and PAFC coagulation were measured at 2 d intervals for the whole storage period up to 10 d, and the results are shown in Fig. 3(a). The content of chlorophyll *a* in the four systems decreased with increasing storage time, indicating that the cell viability decreased. Because of the obvious protection that flocs provide to *M. aeruginosa* cells^19^,^20^,^21^, the decrease of chlorophyll *a* in the systems with coagulation was lower than that in the system without coagulation (*P* < 0.05). Moreover, as noted in Fig. 3(a), the content of chlorophyll *a* in AlCl_3_ and FeCl_3_ sludges was 2.16 ± 0.06 mg/L and 2.31 ± 0.13 mg/L when stored for 10 d, respectively, however, the chlorophyll *a* concentration still remained at 2.97 ± 0.04 mg/L in PAFC sludge after the same storage time, indicating that the cell viability varied in the following sequence when stored for 10 d: PAFC sludge > AlCl_3_ sludge ≈ FeCl_3_ sludge. As we know, chlorophyll *a* auto-fluorescence analysis can show the cell viability directly, hence, auto-fluorescence of *M. aeruginosa* was detected in the four systems (Figure S1). Furthermore, to quantify the fluorescence intensity, the mean fluorescence intensity of cells, which was calculated using total fluorescence intensity divided into the number of cells in each image in Figure S1, in different samples was calculated using Image J program (NIH, USA, Version: ij150-win-jre6-32-bit) and the results are shown in Fig. 3(b). It can be observed that the fluorescence intensity decreased as the storage time prolonged in the four systems, and the decrease of fluorescence intensity in the system without coagulation was higher than that in the systems with coagulation, especially after 6-d storage (*P* < 0.05). Furthermore, it can also be observed that the cell viability varied in the following sequence when stored for 10 d: PAFC sludge > AlCl_3_ sludge ≈ FeCl_3_ sludge, which is in line with the results of chlorophyll *a* described above.

It is well known that chlorophyll *a* content is an important factor in determining the photosynthetic rates. To verify the variation of the chlorophyll *a*, the activity of the other two important photosynthetic enzymes, RuBisCO and PEPCase, was investigated during the storage period. RuBisCO is a rate-limiting enzyme in the photosynthetic carbon reduction cycle and catalyzes the first step of the carbon assimilation process^30^, while PEPCase plays a key role during C_4_ photosynthesis^31^. As shown in Fig. 3(c), the activity of RuBisCO in the four systems also decreased with increasing storage time and the RuBisCO activity in coagulated *M. aeruginosa* cells was higher than that in the system without coagulation (*P* < 0.05), in agreement with the observed variation of chlorophyll *a*. Furthermore, the RuBisCO activity in PAFC coagulation sludge was higher than in AlCl_3_ and FeCl_3_ coagulation sludges after storage for 10 d (*P* < 0.05), consistent with the results for chlorophyll *a* above. In addition, the change of PEPCase activity in the four systems during the storage time was also measured (Fig. 3(d)), and the results were similar to those for the RuBisCO.

**Figure 4:**
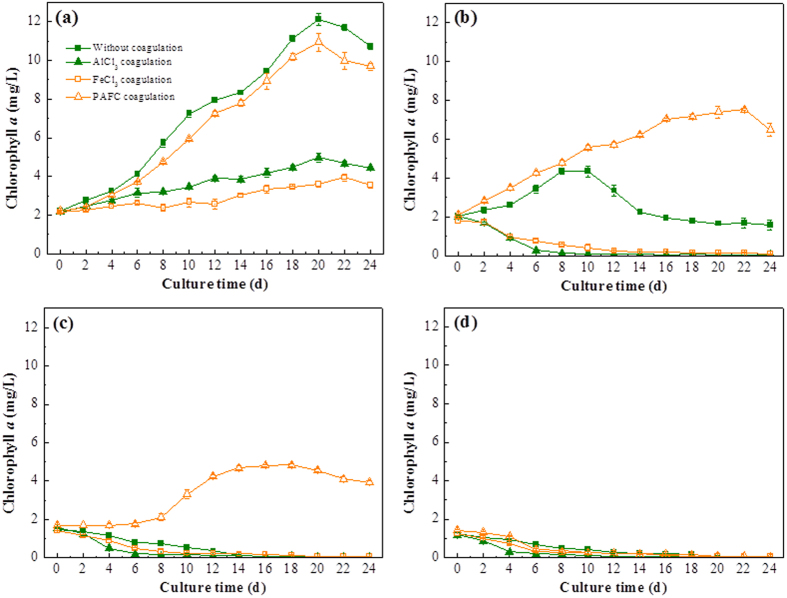
Chlorophyll *a* concentrations in the four systems when the flocs were re-suspended and cultured after storage times of (**a**) 0 d, (**b**) 4 d, (**c**) 6 d, (**d**) 8 d. Data are shown as the mean ± SD (n = 3).

To further test the effects of coagulant species on *M. aeruginosa* cell viability during the sludge storage process, a re-suspended culture experiment was conducted. After the cyanobacteria-containing sludge was stored for a period of time (0, 4, 6, and 8 d), the cyanobacteria-containing sludge was re-suspended and then cultured for 24 d, the results are given in Fig. 4(a–d). As shown in Fig. 4(a), the chlorophyll *a* of the system without coagulation gradually increased from 2.2 ± 0.02 mg/L to 12.1 ± 0.33 mg/L within 20 days’ incubation and then decreased with increasing culture time. Because the growth of *M. aeruginosa* may be inhibited, in part, by the flocs, the chlorophyll *a* level in the PAFC-coagulated system increased from 2.2 ± 0.03 mg/L to 10.9 ± 0.45 mg/L within the tested 20 days, however, the chlorophyll *a* of the systems with AlCl_3_ and FeCl_3_ coagulation only rose from 2.2 ± 0.03 mg/L to 5 ± 0.25 mg/L and 3.6 ± 0.15 mg/L, respectively, when the systems were cultured for 20 days. Figure 4(b) shows the growth of *M. aeruginosa* cells which were resuspended after the sludge was stored for 4 d. The cell viability in the uncoagulated system has obviously declined, and the chlorophyll *a* level only reached 4.37 ± 0.16 mg/L (the maximum level) after 8 d of culturing, and then declined. However, chlorophyll *a* content with PAFC coagulation increased to 7.52 ± 0.06 mg/L within 22 days’ culturing. In contrast, the concentration of chlorophyll *a* with AlCl_3_ and FeCl_3_ coagulation rapidly declined to 0 within 10 days’ incubation. Figure 4(c) further verified these results. Despite the sludge having been stored for 6 d, the *M. aeruginosa* cells in PAFC flocs were still viable and chlorophyll *a* concentration rose to 4.83 ± 0.07 mg/L after 18 days’ incubation. When the sludge had been stored for 8 d, the cell viability was further decreased as shown before (see Fig. 3(a)). Due to the low viability of *M. aeruginosa* cells after storage for 8 d, the *M. aeruginosa* cells cannot regrow and the chlorophyll *a* content rapidly decreased to 0 after culturing the resuspended material for a few days (Fig. 4(d)). Therefore, these results confirmed the conclusion that the cell viability in PAFC sludge was higher than FeCl_3_ and AlCl_3_ sludges, and the cell viability in AlCl_3_ sludge was similar to FeCl_3_ sludge for the same storage time.

### The relationship between cell integrity and cell viability during sludge storage

Interestingly, the cell viability of *M. aeruginosa* cells in sludge varied as follows for the same storage time: AlCl_3_ sludge ≈ FeCl_3_ sludge < PAFC sludge, whereas the lysis of *M. aeruginosa* cells varied in the following sequence at the same storage time: AlCl_3_ sludge > FeCl_3_ sludge ≈ PAFC sludge. Though the cell viability of *M. aeruginosa* in FeCl_3_ sludge gradually declined with increased storage time, there was basically no breakage of algae cells for up to 10 d of storage. These results may be related to the different properties of the flocs, such as floc size and density, produced by different types of coagulants.

**Figure 5:**
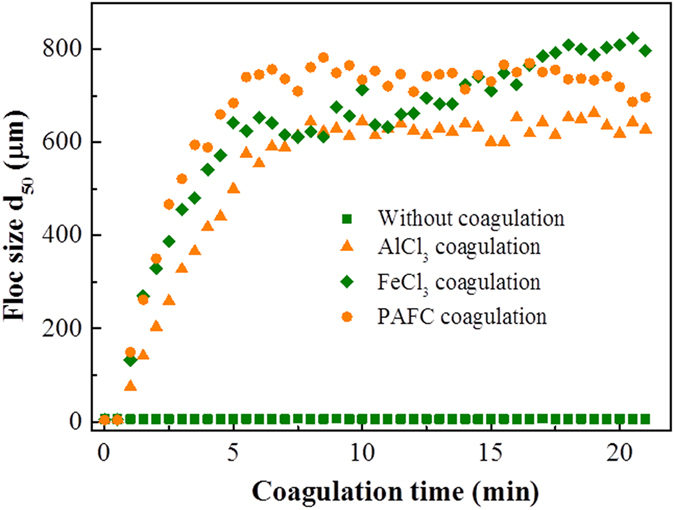
The formation and growth of *M. aeruginosa* flocs formed by AlCl_3_, FeCl_3_, and PAFC during the coagulation process.

To test this hypothesis, the characteristics of the flocs during the coagulation process were evaluated and the results are summarized in Fig. 5 and Table 1. The initial median diameter (*d*_50_) in the bloom water was about 5 μm, and grew rapidly once coagulants were dosed (Fig. 5). AlCl_3_ and PAFC flocs grew rapidly in the first 5 minutes and then remained stable, and the final *d*_50_ values of the flocs were about 618 μm and 719 μm, respectively. In contract, the flocs formed by FeCl_3_ gradually grew to become much larger (805 μm) than those of AlCl_3_ and PAFC. Therefore, the better protective effect of FeCl_3_ flocs might be partly due to the larger floc size.

In addition, the fractal structure of flocs was analyzed through the measurement of fractal dimension (*D*_f_), which could be obtained by the exponential relationship of mass and particle size^32^ and could indicate the development of aggregate structure during the formation of flocs^33^. As presented in Table 1, the fractal dimension of AlCl_3_, FeCl_3_ and PAFC flocs were 1.88, 2.24, and 1.79, respectively. Bridgeman *et al*.^34^ reported that higher *D*_f_ values indicated more compact structures, while flocs with lower *D*_f_ values were looser and more open. Therefore, these results demonstrated that FeCl_3_ flocs were more compact than AlCl_3_ and PAFC flocs, which also indicated FeCl_3_ flocs have a better protective effect on the *M. aeruginosa* cells than do AlCl_3_ and PAFC flocs. Hence, although the cell viability of *M. aeruginosa* in FeCl_3_ sludge was lower than that in PAFC sludge, there was basically no damage of algae cells for up to 10 d of storage (similar to PAFC sludge).

### Proposed mechanisms involved in the actions of coagulant species on *M. aeruginosa* cells during sludge storage

As discussed above, the *M. aeruginosa* cell viability varied in the following sequence for equal storage times: AlCl_3_ sludge ≈ FeCl_3_ sludge < PAFC sludge. The initial pH value has been identified as an important factor affecting the growth and cell viability of algae^35^,^36^, and Qian *et al*.^36^ reported that low-pH stress (pH < 5) could cause the lysis of algae cells and metabolite release. To explore whether our result is related to the initial pH value, we examined the initial pH value in the four systems after coagulation, and the results are shown in Table 1. As noted in Table 1, the pH value of the bloom water was 8.49 ± 0.05, and due to the hydrolysis of Fe^3+^ and Al^3+^ during the coagulation process, the pH value decreased to 8.02 ± 0.04, 7.42 ± 0.1, and 8.35 ± 0.06 after AlCl_3_, FeCl_3_, and PAFC coagulation, respectively. McLachlan and Gorham^37^ reported that *M. aeruginosa* could grow well in the pH range of 6.5–10. Therefore, the pH value should not be the main factor affecting the cell viability in this study.

In addition, it is well known that Al is toxic to freshwater algae^38^,^39^,^40^, and Gensemer and Playle^40^ reported that aluminum likely reduces P uptake rates in algae, perhaps by inhibition of the acid phosphatase enzyme. Because iron is a key component of chromophore synthesis, and biosynthesis of chlorophyll and phycobilin pigments includes iron-dependent steps even though neither of them contain iron^41^, iron is usually essential for cell growth, for example, Imai *et al*.^42^ and Wang *et al*.^43^ reported that the growth rate of *M. aeruginosa* increased with increasing Fe concentration when the concentration of Fe was low. However, the growth would obviously be inhibited when the Fe concentration was higher than 1377 μg/L^43^. Furthermore, Kumawat *et al*.^44^ reported that iron affects chlorophyll synthesis indirectly by affecting its precursor, δ-aminolevulinic acid (ALA), thereby affecting the growth of algae cells. To explore whether the concentrations of Al and Fe influence the activity of *M. aeruginosa* cells during storage, we analyzed Al and Fe concentrations after coagulation (Table 1). The content of Al and Fe in the bloom water before coagulation was about 28.6 ± 2.3 μg/L and 11.7 ± 3.9 μg/L, respectively (Table 1). Correspondingly, the concentration of Al increased to 705 ± 26.4 μg/L after 15 mg/L AlCl_3_ coagulation. According to the U.S. Environmental Protection Agency (US-EPA), total Al concentrations ranging from 460 μg/L to 6480 μg/L are toxic to freshwater algae^40^,^45^. Therefore, the high level of Al observed (705 ± 26.4 μg/L) is hazardous to the growth of *M. aeruginosa* in our study. Because 50 mg/L FeCl_3_ was added, the Fe concentration drastically increased to 2999 ± 35.4 μg/L after coagulation, higher than 1377 μg/L, which could obviously inhibit *M. aeruginosa* growth. Moreover, the Al concentration only increased to 240.2 ± 15.1 μg/L after 15 mg/L PAFC was added, which has little or no toxic effect on *M. aeruginosa* cells, whilst the concentration of Fe increased to 80.7 ± 7.4 μg/L, which may be of benefit to *M. aeruginosa* growth.

To further test the effect of Al and Fe concentration on *M. aeruginosa* cells during storage, the physiological characteristics of *M. aeruginosa* cells were investigated. Wang *et al*.^46^ and Xia *et al*.^47^ reported that the intracellular reactive oxygen species (ROS) would be increased in *M. aeruginosa* cells after treatment by either CuO or TiO_2_ nanoparticles. Furthermore, superoxide radicals and hydrogen can be consumed by SOD to produce hydrogen peroxide and oxygen, which has been described as the first line of resistance against ROS^48^. Thus, to explore whether high concentrations of Fe or Al could induce the increase of ROS in *M. aeruginosa* cells, ROS level and the responses of SOD were investigated at 2 d intervals during the whole sludge storage time up to 10 d.

**Figure 6:**
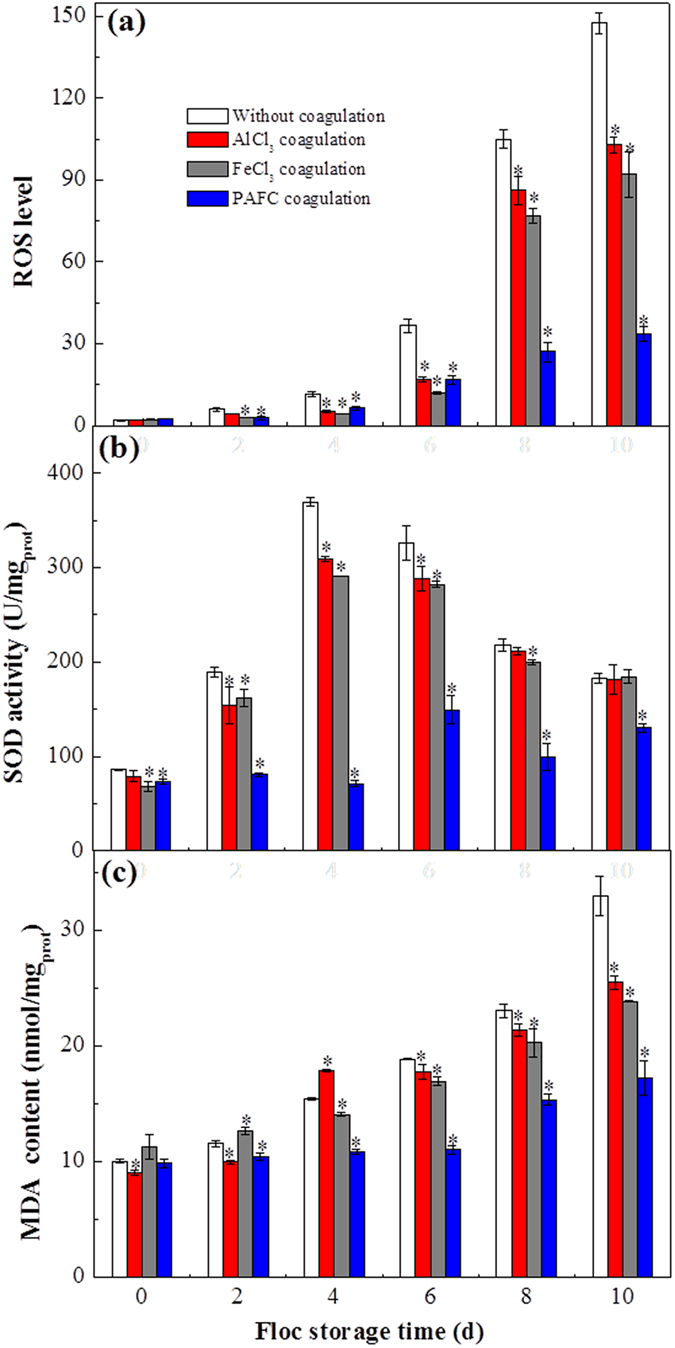
ROS level (**a**), SOD activity (**b**) and MDA content (**c**) of the *M. aeruginosa* cells in the four systems at different floc storage times (0, 2, 4, 6, 8, and 10 d). Data are shown as the mean ± SD (n = 3). Asterisks above the bars indicate significant differences with respect to the system without coagulation (*P* < 0.05).

As shown in Fig. 6(a), the level of ROS increased dramatically as the storage time prolonged in the four systems, and the increase of ROS in the systems with coagulation was significantly lower than that in the system without coagulation (*P* < 0.05), owing to the protection of flocs. Moreover, it is notable that the increase of ROS in the system with PAFC coagulation was obviously lower than that in the systems with AlCl_3_ and FeCl_3_ coagulation (*P* < 0.05). The reason may be due to the toxicity of Fe and Al ions aforementioned.

SOD activity of *M. aeruginosa* increased initially and then decreased as storage time was extended (Fig. 6(b)). The increase of SOD in the initial stage might be ascribed to the initial stresses, such as the toxicity of Fe and Al ions, and the subsequent SOD decrease implies that the enhanced ROS level had exceeded the scavenging ability of the antioxidant enzymes^49^. Due to the protection of flocs, the increase of SOD in coagulated systems was lower than in the uncoagulated system (*P* < 0.05). In addition, the SOD activity reached 309.7 ± 2.36 and 291.6 U/mg_prot_ (the maximum level) on the fourth day with AlCl_3_ and FeCl_3_ coagulation, respectively, whereas the SOD activity only reached 149.5 ± 14.9 U/mg_prot_ (the maximum level) on the sixth day with PAFC coagulation, which means that the ROS level in *M. aeruginosa* cells with AlCl_3_ and FeCl_3_ coagulation was more severe than in the cells with PAFC coagulation. Therefore, from the result above, we verified that high concentrations of Al and Fe were harmful to the growth of *M. aeruginosa* cells. Furthermore, a moderate Fe level was beneficial to *M. aeruginosa* growth, and low level of Al has no effect on *M. aeruginosa* cells.

MDA, one of several low-molecular-weight end products formed by the decomposition of polyunsaturated fatty acid hydroperoxides, is usually used as a biomarker of physiological stresses and cellular oxidative damage^50^. To verify the results above (Fig. 6(a,b)) and further investigate whether the oxidative stress, such as toxicity of Al and Fe at high concentrations, could cause oxidative damage on *M. aeruginosa* cells, the variation of MDA was examined (Fig. 6(c)). As shown in Fig. 6(c), the MDA content increased with increasing storage time, and the MDA content in *M. aeruginosa* cells with coagulation was lower than that in the system without coagulation (*P* < 0.05), which illustrated that the cellular oxidative damage in *M. aeruginosa* cells with coagulation was lower than that in the system without coagulation, and the reason might be attributed to the protection of the flocs. As storage time increased, it was found that the MDA content with PAFC coagulation was lower than that with AlCl_3_ or FeCl_3_ coagulation (*P* < 0.05), indicating that the cellular oxidative damage in *M. aeruginosa* cells with AlCl_3_ or FeCl_3_ coagulation was more severe than in the cells with PAFC coagulation. In addition, due to the FeCl_3_ flocs being larger and more compact than AlCl_3_ flocs, there was a stronger protective effect for FeCl_3_ flocs than AlCl_3_ flocs. Hence, the MDA content in *M. aeruginosa* cells with FeCl_3_ coagulation was slightly lower than that with AlCl_3_ coagulation for equal storage times, indicating that the cellular oxidative damage in the *M. aeruginosa* cells of FeCl_3_ sludge was lower than that in AlCl_3_ sludge.

**Figure 7:**
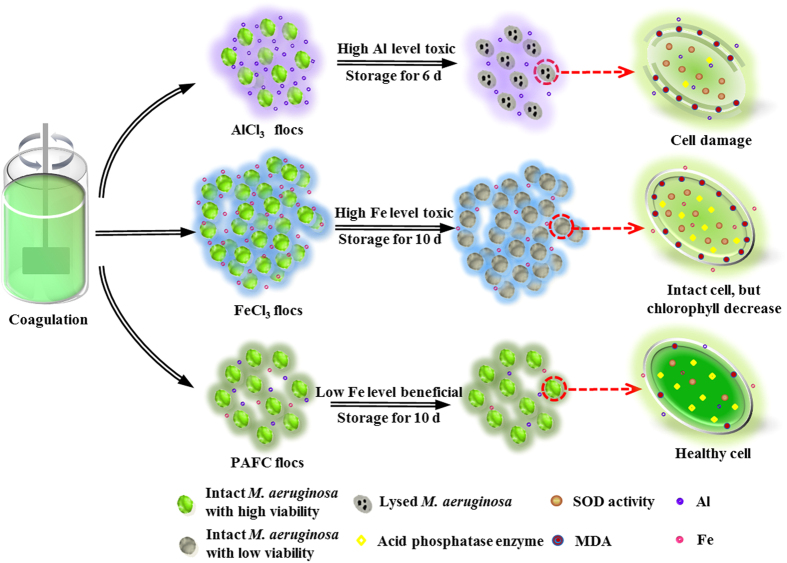
Schematic diagram depicting the effect of AlCl_3_, FeCl_3_, and PAFC on *M. aeruginosa* cell viability and integrity during storage of cyanobacteria-containing sludges.

Based on the discussion above, due to the protection of flocs, the integrity and viability of *M. aeruginosa* cells with coagulation was better than for uncoagulated cells. Furthermore, coagulant species have an obvious effect on cell integrity and viability during sludge storage. As shown in Fig. 7, for cyanobacteria-containing AlCl_3_ and FeCl_3_ sludges, the high levels of Al and Fe may inhibit the activity of acid phosphatase enzyme and hinder the synthesis of chlorophyll in *M. aeruginosa* cells, respectively. The higher floc size and density for FeCl_3_ flocs may restrict the absorption of light for *M. aeruginosa* cells, which also prevents the synthesis of chlorophyll for cells in FeCl_3_ flocs. Thus the normal metabolism of *M. aeruginosa* cells would be destroyed. Furthermore, due to the adverse environmental stressors, such as high concentration of Al and Fe, excess ROS may be produced in *M. aeruginosa* cells with increased storage time. Because SOD was the first line of resistance against ROS, SOD activity was then induced in response to the oxidative stress. However, when ample ROS was produced in *M. aeruginosa* cells, the activity of SOD would be limited and the cell membrane would be damaged by lipid peroxidation. Then the viability of *M. aeruginosa* cells would be decreased and the integrity of the cell membrane would be damaged. More suitable Fe concentrations could promote the growth of *M. aeruginosa* cells in cyanobacteria-containing PAFC sludge, whilst low concentration of Al has little or no effect on *M. aeruginosa* cells. There was no excess ROS accumulated in *M. aeruginosa* cells, and basically no lipid peroxidation on the cell membrane, hence, the *M. aeruginosa* cells maintained relatively high activity and integrity. Correspondingly, the *M. aeruginosa* cells in AlCl_3_ sludge began to lyse after 6 d of storage and the *M. aeruginosa* cells in PAFC sludge remained intact after up to 10 d storage. However, the *M. aeruginosa* cells in FeCl_3_ sludge also remained intact after up to 10 d storage (similar to PAFC sludge). The reason may be ascribed to the higher floc size and density for FeCl_3_ flocs, which can more effectively prevent the damage of *M. aeruginosa* cells.

## Conclusions

In summary, coagulant species have a significant effect on the cell viability and integrity of *M. aeruginosa* cells during sludge storage processes. Because high concentrations of Al and Fe are toxic to *M. aeruginosa*, while appropriate amount of Fe is beneficial to the growth of *M. aeruginosa,* the *M. aeruginosa* cells in PAFC sludge have higher cell viability than those in AlCl_3_ and FeCl_3_ sludges after the same storage times. Furthermore, we also found that the lysis of *M. aeruginosa* cells in AlCl_3_ sludge occurred earlier and was more severe than the *M. aeruginosa* cells in the systems with FeCl_3_ and PAFC coagulation. Consistent with the protective function of the large floc size and high density of the FeCl_3_ sludge, the *M. aeruginosa* cells remained basically intact in that sludge after 10 d storage, even though the cell viability was low. Hence, FeCl_3_ may be an ideal coagulant that can not only reduce the viability of *M. aeruginosa* cells but also prevent the lysis of the cells. Overall, this study provided not only a systematic analysis of *M. aeruginosa* cells’ death and lysis in AlCl_3_, FeCl_3_ and PAFC sludges upon increasing storage time, but also guidance for the safe treatment of coagulation sludges in drinking water treatment plants.

## Materials and Methods

### Algal culturing

*Microcystis aeruginosa (M. aeruginosa*, FACHB-905, obtained from the Institute of Hydrobiology, Chinese Academy of Sciences) was selected as the experimental cyanobacteria, and was grown in BG11 medium (pH 7.5). The cultures were maintained in a constant temperature incubator (GXZ-280C, Ningbo Jiangnan Instrument Factory, China) at 25 °C under 2000 lux with a light–dark cycle of 12 h/12 h and harvested at the late exponential phase of growth with a final cell yield up to about 10^7^ cells/mL.

### Raw water and bloom water

The raw source water investigated in this research was sampled from the Queshan Reservoir, one of the important drinking water sources in Jinan, China, and filtrated through a 0.45 μm cellulose acetate membrane (Shanghai Mili Membrane Separation Technology Co., China) to remove natural algae. The main characteristics of the raw water were: temperature 16.7 °C, pH 8.4, turbidity 1.31 NTU, UV_254_ 0.047 cm^−1^, DOC 4.67 mg/L, NH_3_-N 0.19 mg/L, TN 2.2 mg/L, TP 0.03 mg/L, alkalinity 133.9 mg/L, residual Al 91.5 μg/L, and residual Fe 58.3 μg/L. To simulate the algal bloom in a high algae-laden period according to the guidance value of WHO^12^, the raw water was spiked with *M. aeruginosa* cultures to achieve a final cell density of about 10^6^ cells/mL (bloom water).

### Chemicals

Ferric chloride hexahydrate (FeCl_3_·6H_2_O, AR grade) and aluminum chloride hexahydrate (AlCl_3_·6H_2_O, AR grade) were purchased from Sinopharm Chemical Reagent Co. (Shanghai, China). Polymeric aluminum ferric chloride (PAFC) was purchased from Gongyi Yongxing Biochemical Materials Co., China. The main parameters of the PAFC were: relative density 1.25 (20 °C), alumina content 8–10%, iron oxide content 1–2%, basicity 65–90%. The AlCl_3_, FeCl_3_ and PAFC stock solutions were prepared to be 3 g/L (pH 3.22), 10 g/L (pH 1.65) and 5 g/L (pH 4.44), respectively.

### Coagulation experiments

Coagulation experiments were performed at room temperature (25 ± 2 °C) in a six-paddle stirrer (ZR4-6, Zhongrun Water Industry Technology Development Co., China). For the coagulation experiment, each bloom water sample (1 L) was dosed with the stock solution when the rapid mixing began. The optimum coagulation conditions for the effective removal of *M. aeruginosa* cells by AlCl_3_, FeCl_3_, and PAFC, respectively, are listed in Table S1. After coagulation, samples were quiescently settled for 30 min to obtain the flocs (formed into sludge) and supernatants.

### Floc storage experiments

After removing 930 mL of supernatant, 70 mL of cyanobacteria-containing sludge remained. For a control (without coagulation), the bloom water sample (1 L) was centrifuged at 6000 rpm for 3 min, and the cells were then re-suspended into the raw water to make up 70 mL, forming a coagulant-free sludge. All cyanobacteria-containing sludge samples were then housed in an incubator at 25 °C under 2000 lux illumination with the 12 h/12 h (light/dark) cycle for up to 10 d. The sludge samples were drawn every two days for analysis during the whole storage period.

### Cell integrity analysis during the storage process

The intracellular materials would be released when the cell membrane is damaged. Therefore, the change of extracellular MCs, polysaccharide, total nitrogen (TN), and total phosphorus (TP) was measured, to investigate the integrity of *M. aeruginosa* cells during storage. The sludge samples were drawn every two days during storage and filtered through 0.45 μm cellulose acetate membranes. Extracellular MC content was measured using a Beacon Microcystin ELISA kit (Beacon Analytical Systems, Maine, USA). The method used for the detection of MCs by ELISA was performed as previously reported^20^. Extracellular polysaccharide concentration was determined by the phenol–sulfuric acid method^51^. Extracellular TN and TP were conducted according to Chinese state standard testing methods^52^.

To directly assess the surface information and morphology of *M. aeruginosa* cells during the storage time, scanning electron microscopy (SEM) was used. The sludge samples were centrifuged at 4000 rpm for 5 min, the pellets were pre-fixed with 2.5% glutaraldehyde overnight, washed by phosphate buffer solution three times and post-fixed with 1% osmium tetraoxide for 1 h, then again washed with phosphate buffer solution. After that, samples were consecutively treated with 50%, 75%, 90% and 100% ethanol solutions (15 min each) and dried with a vacuum drier. The completely dry samples were then mounted on a copper stub, coated with gold and examined with an SEM (S-4100, Hitachi, Japan) at 3 kV.

### Cell viability analysis during the storage process

To explore the change of cell viability during the sludge storage period, chlorophyll *a* content, chlorophyll *a* auto-fluorescence, activity of Ribulose-1,5-bisphosphate carboxylase (RuBPCase) and Phosphoenol-pyruvate carboxylase (PEPCase) in *M. aeruginosa* were measured. The chlorophyll *a* content was measured according to the method of Pancha *et al*.^53^. A fluorescence microscope (NIKON TE2000, Japan) fitted with filters including exciter filter EX510-560, dichroic mirror DM575, barrier filter BA590, was used for chlorophyll *a* auto-fluorescence observation. The red emission spectra were captured using a CCD camera. Activity of RuBPCase and PEPCase in *M. aeruginosa* cells was measured by utilizing the plant RuBPCase ELISA Kit (R&D, USA) and the plant PEPCK ELISA kit (R&D, USA), respectively. The activity of RuBPCase and PEPCase in *M. aeruginosa* cells were expressed as the ratios of RuBPCase and PEPCase to intracellular protein (pg/mg_prot_), respectively.

In addition, to further study the viability of the *M. aeruginosa* cells in the sludge formed by AlCl_3_, FeCl_3_, and PAFC after different storage time, a re-suspended culture experiment was conducted according to the method of Li and Pan^54^. After storage of the AlCl_3_, FeCl_3_, and PAFC cyanobacteria-containing sludge for 0, 4, 6, and 8 d, 70 mL of BG11 medium (double concentrated) was added to each sludge sample. The re-suspended cyanobacteria-containing sludge samples were maintained in the illuminated incubator at 25 °C under 2000 lux illumination on the 12 h/12 h (light/dark) cycle. The recovery and regrowth of the *M. aeruginosa* cells were monitored by detecting the chlorophyll *a* concentration in the culture over the following 24 days.

### Floc properties, pH, Al and Fe during the coagulation process

A laser diffraction instrument, Mastersizer 2000 (Malvern, UK), was used to monitor dynamic floc size. Size measurements of the flocs were taken every 30 s in the process of coagulation and the results were recorded automatically by the computer. The size data were expressed as equivalent volumetric diameters, and the floc size was characterized by the median volumetric diameter (*d*_50_).

The fractal dimension (*D*_f_) of aggregates was measured by a small-angle laser light scattering (SALLS) method by the Mastersizer 2000 with a 632.8 nm laser light beam^55^. The total scattered light intensity *I*, the scattering vector *Q*, and *D*_f_ followed a power law as shown in Eq. (1)^55^:


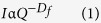


The scattering vector *Q* is the difference between the incident and scattered wave vectors of the radiation beam in the medium as shown in Eq. (2)^56^:


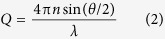


where *n, θ* and *λ* are the refractive index of the medium, the laser light wavelength in vacuum, and the scattering angle, respectively.

Furthermore, after coagulation, the pH of the bloom water was measured using a digital pH-meter (pHS-3C, Leici, China). The concentration of residual aluminum and iron in the bloom water and the cyanobacteria-containing sludge were determined using an inductively-coupled plasma optical emission spectrometer (180-80, Hitachi, Japan). Samples were filtered through 0.45 μm cellulose acetate membranes and acidified to pH < 2 with HNO_3_, and digested for 2 h by a Lianhua 5B-1B digestion device (Lianhua Tech. Co., Beijing, China) before analysis.

### ROS, SOD and MDA analysis in *M. aeruginosa* cells during the storage process

The reactive oxygen species (ROS) level in *M. aeruginosa* cells during storage was determined as previously reported^57^. The ROS level in the presence of algae was expressed as the ratio of fluorescence emission intensity to intracellular protein.

To extract antioxidation enzymes, 2 mL of each sludge sample were centrifuged at 8000 rpm for 10 min. The cell pellets were then re-suspended with phosphate buffered saline solution (50 mM, pH 7.0) and homogenized by an ultrasonic cell pulverizer (Scientz-IID, China) at 600 W for a total time of 10 min (cycle: 2 s ultrasonication, 8 s rest) in an ice bath. Finally, the homogenate was centrifuged at 12000 rpm for 10 min at 4 °C to obtain the supernatant for assays of the enzyme activity and the level of lipid peroxidation. Superoxide dismutase (SOD) activity in *M. aeruginosa* cells was determined by the inhibition of nitro blue tetrazolium reduction according to the method of Beauchamp and Fridovich^58^. Malondialdehyde (MDA) content in algal cells was measured from material reacting to thiobarbituric acid, according to the thiobarbituric acid reaction as described by Dogru *et al*.^59^. MDA and SOD results were expressed as μmol/mg_prot_ and U/mg_prot_, respectively. Furthermore, the intracellular protein was determined by the method of Bradford^60^ using bovine serum albumin as a standard and the results are shown in Figure S2.

### Statistical analysis

All experiments performed were performed in triplicate and the data were expressed as the means ± standard deviation (SD). All of the parameters were compared across treatments with one-way ANOVA using the SPSS software (version 16.0), and the significance was set to *P* < 0.05. All statistical analyses were carried out using Origin 9.1.

## Additional Information

**How to cite this article**: Xu, H. *et al*. Behaviors of *Microcystis aeruginosa* cells during floc storage in drinking water treatment process. *Sci. Rep.* **6**, 34943; doi: 10.1038/srep34943 (2016).

## Supplementary Material

Supplementary Information

## Figures and Tables

**Table 1:** The fractal dimension, pH, and Al and Fe concentrations before coagulation and during the AlCl_3_, FeCl_3_, and PAFC coagulation processes.

	*D*_f_	pH	Al concentration(μg/L)	Fe concentration(μg/L)
Without coagulation	No data	8.49 ± 0.05	28.6 ± 2.3	11.7 ± 3.9
AlCl_3_ coagulation	1.88	8.02 ± 0.04	705 ± 26.4	12.1 ± 5.5
FeCl_3_ coagulation	2.24	7.42 ± 0.1	32.6 ± 4.2	2999 ± 35.4
PAFC coagulation	1.79	8.35 ± 0.06	240.2 ± 15.1	80.7 ± 7.4

Data are shown as the mean ± SD (n = 3).
