# Supplementary material for: Behaviors of Microcystis aeruginosa cells during floc storage in drinking water treatment process
Source: Sci Rep. 2016 Oct 7;6:34943. doi: 10.1038/srep34943 (PMC5054690; doi:10.1038/srep34943)
Supplement: Supplementary Information [file srep34943-s1.pdf]

Supplementary Information

Behaviors of *Microcystis aeruginosa* cells during floc storage in drinking water treatment process

Hangzhou Xu[1], Haiyan Pei[1, 2]*, Hongdi Xiao[3], Yan Jin[1], Xiuqing Li[1], Wenrong Hu[1, 2], Chunxia Ma[1] , Jiongming Sun[1], Hongmin Li[1]

[1] *School of Environmental Science and Engineering, Shandong University, Jinan, 250100, China.*

[2] *Shandong Provincial Engineering Center on Environmental Science and Technology, Jinan, 250061, China.*

[3] *School of Physics, Shandong University, 250100, China.*

* Corresponding author: School of Environmental Science and Engineering, Shandong University, Jinan 250100, P. R. China

Tel./Fax: +86-531-88392983

E-mail address: haiyanhup@126.com.

Supplementary Figures

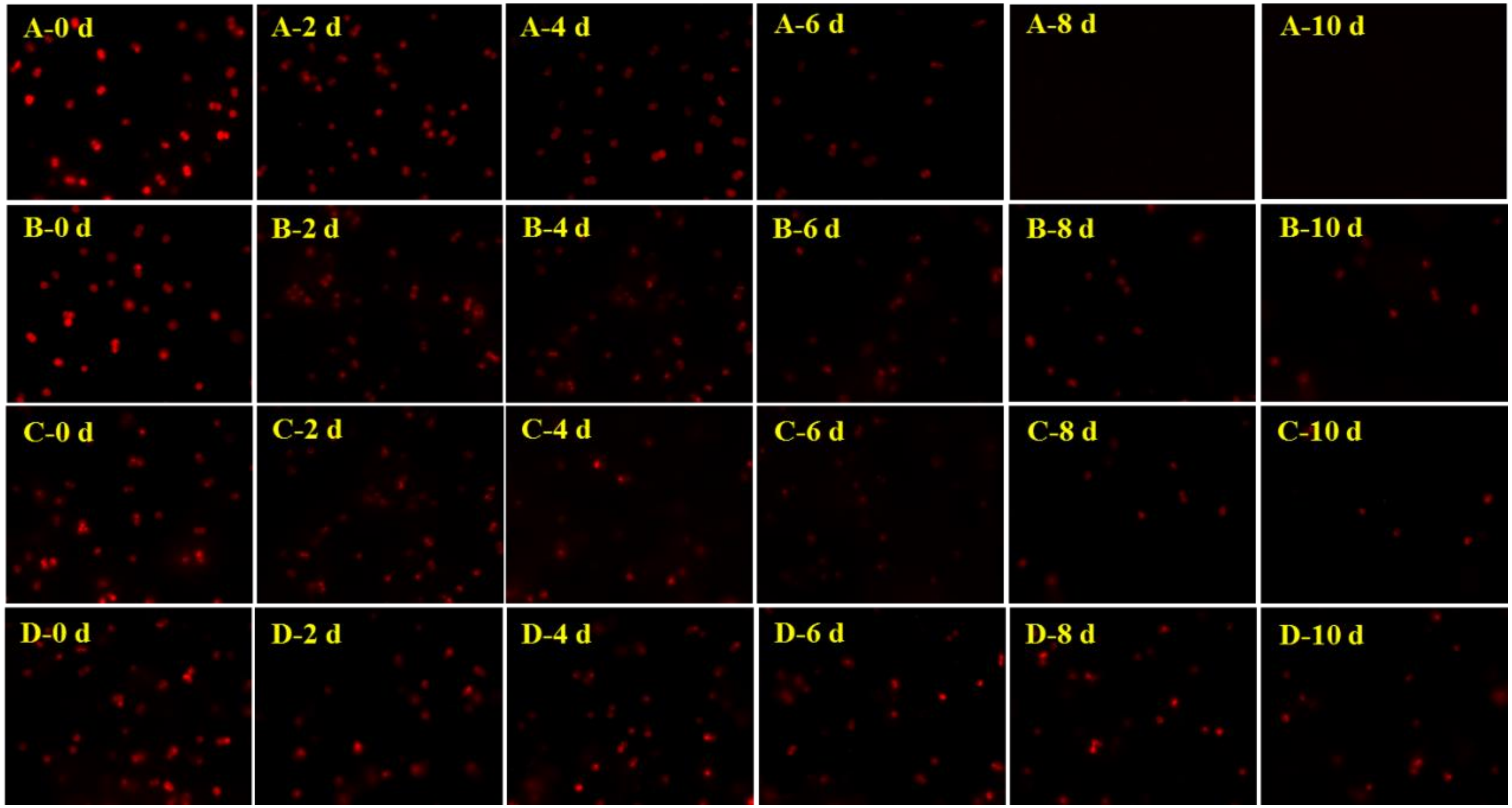

Figure S1 Chlorophyll *a* autofluorescence of the *M. aeruginosa* cells in the flocs in the four systems at different floc storage times (0, 2, 4, 6, 8, and 10 d). (A: without coagulation, B: $AlCl_3$ coagulation, C: $FeCl_3$ coagulation, and D: PAFC coagulation, respectively.)

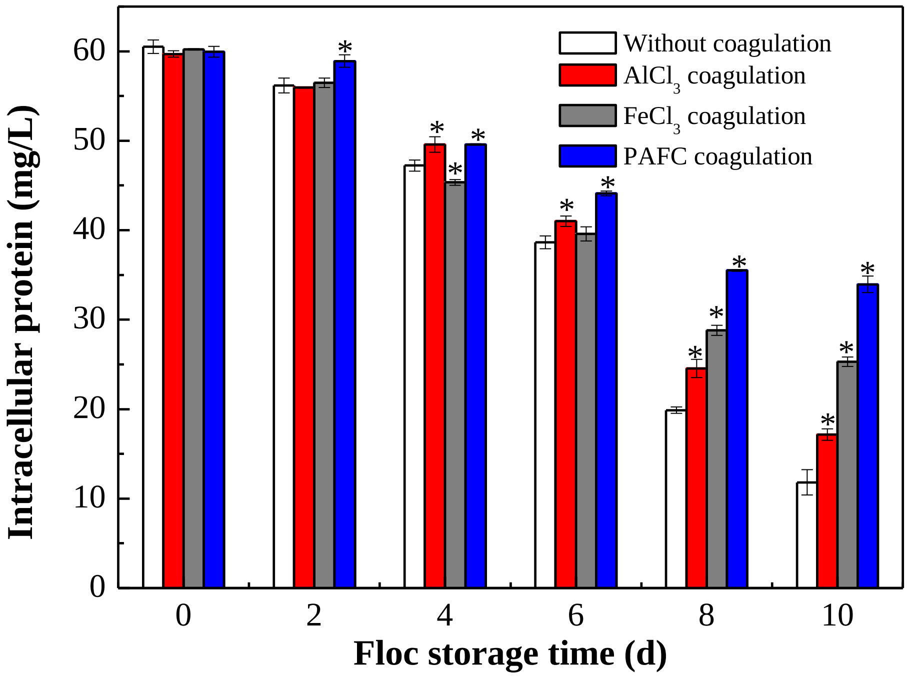

Figure S2 Intracellular protein concentrations in the four systems at different floc storage times (0, 2, 4, 6, 8, and 10 d). Data are shown as the mean ± SD (n=3). Asterisks above the bars indicate significant differences with respect to the system without coagulation ($P<0.05$)

Supplementary Table

Table S1 The optimum values of coagulation conditions for the removal of *M. aeruginosa* cells by $AlCl_3$, $FeCl_3$, and PAFC, respectively.

Coagulant species	Coagulant dosage (mg/L)	Rapid mixing		Slow mixing		Reference
		Speed (rpm)	Time (min)	Speed (rpm)	Time (min)	
$AlCl_3$	15	250	1	20	20	Sun et al., 2012
$FeCl_3$	50	250	0.5	20	30	Li et al., 2015
PAFC	15	250	1	40	20	Xu, 2015
